# Editorial: Somatic mutations, genome mosaicism and aging

**DOI:** 10.3389/fragi.2022.1115408

**Published:** 2023-01-09

**Authors:** Michael A. Lodato, Jan Vijg

**Affiliations:** ^1^ Department of Molecular, Cell, and Cancer Biology, University of Massachusetts Chan Medical School, Worcester, MA, United States; ^2^ Center for Single-Cell Omics, School of Public Health, Shanghai Jiao Tong University School of Medicine, Shanghai, China; ^3^ Department of Genetics, Albert Einstein College of Medicine, New York City, NY, United States

**Keywords:** somatic mutation, aging, genome mosaicism, DNA damage, oxidative stress

## Introduction

We present a Research Topic entitled “*Somatic mutations, genome mosaicism, and aging*” in Frontiers in Aging. Somatic mutations are defined as mutations that occur in cells of the body after fertilization. Since they are the root cause of essentially all cancers, somatic mutations have been extensively studied in oncogenesis. However, somatic mutations can and do occur in non-cancerous cells at any stage of embryonic development, after birth, and during aging. Somatic mutations result in genome mosaics, with cells of the same organism having distinct genome sequences. Because of this mosaicism, with most mutations unique for each individual cell, somatic mutations have been challenging to study, necessitating the development of specialized molecular and bioinformatic tools to characterize them. During aging, patterns of somatic mutation and genome mosaicism change, potentially impacting the steady decline in normal function and increase in susceptibility to disease that defines the aging process. The reviews in this collection cover a wide array of sub-topics, including the causes of somatic mutation during aging, the consequences of genome mosaicism, and the molecular and bioinformatic tools that can be used to identify somatic mutations (Table 1). In this editorial, we summarize the major themes of each review article.

**TABLE 1:** Scientific Areas covered in this Research Topic, and the individual articles in which these areas are discussed. Additional review material published outside of this Research Topic are also listed for further reading.

Scientific area	References in this research topic	Additional references
Bioinformatic analysis of somatic mutations	Huang and Lee	Valecha and Posada, (2022)
Technologies for mosaic variant identification	Walsh et al., Ren et al., Manders et al., Sanchez-Contreras and Kennedy, Huang and Lee	Dou et al. (2018)
Clonal Hematopoiesis	Walsh et al.	Jaiswal and Ebert, (2019)
Somatic mutations and aging	Walsh et al., Ren et al., Manders et al., Sanchez-Contreras and Kennedy	Miller et al. (2021)
Causes of somatic mutation and mutation signatures	Ziegenfuss and Lodato, Ren et al., Manders et al., Walsh et al., Huang and Lee	Koh et al. (2021)
Somatic mutations in mitochondrial genomes	Sanchez-Contreras and Kennedy	Smith et al. (2022)
Tissue- and Cell-Type-Specific Somatic Mutagenesis	Ren et al., Manders et al.,	
Animal models of somatic mutation	Contreras and Kennedy, Walsh et al.	Franco et al., 2019
		Wang et al. (2022)
Oxidative DNA damage and somatic mutations	Ziegenfuss and Lodato, Sanchez-Contreras and Kennedy	Biswas et al. (2022)

## Main text

Huang and Lee review the current state of bioinformatic tools to study somatic mutations. They outline methods to identify somatic mutations in two types of data. First, sequencing of “bulk” DNA, which is DNA harvested after the lysis of tissue samples comprising millions of cells, and, second, single-cell sequencing, whereby the genomes of individual cells are sequenced, generally after an amplification step. Bulk DNA somatic mutation calling strategies grew from the field of cancer genomics, where somatic mutations are defined using differential allele fractions in tumor and normal tissue. Heuristics and machine-learning filters have been applied in more recent algorithms utilizing this framework. Single-cell sequencing represents the highest level of resolution possible for somatic mutation analysis, but artifacts generated during whole-genome amplification prior to sequencing required the development of specialized tools to reliably identify somatic mutations in these sequencing data. These tools unlocked the amazing potential of single-cell sequencing to use developmental somatic mutations as natural lineage tags, and to analyze somatic mutagenesis during aging.

Manders et al. review the dynamics of somatic mutagenesis during human life. They provide an impressive survey of over twenty studies analyzing somatic mutation accumulation in adult tissues during aging. Collectively, these studies demonstrate that all tissues analyzed accumulate somatic mutations linearly over time, but at variable rates across tissues and across cells within tissues. Manders et al. also discuss how the analysis of *mutational signatures*, which are patterns of the types of nucleotide changes that comprise the set of somatic mutations in a sample, can identify mutagenic and repair pathways involved in generating somatic mutations. Such signatures have identified hallmarks of DNA damage in somatic mutation datasets from various tissues by mutagens such as UV light, alkylating agents, alcohol, endogenous deaminases of the APOBEC family, and stochastic base decay. Somatic mutation rates sometimes differ between terminally differentiated cells and the stem/progenitor cells that generate them, but this phenomenon is not consistent. However, somatic mutation accumulation rates are consistently elevated in prenatal cells relative to postnatal cells, potentially owing to the rapid and possibly error-prone cell divisions of the preimplantation embryo.

Ren et al. review the analysis and potential impact of somatic mutation burden in human tissues during aging. They point out that somatic mutations happen because there are benefits but also costs to keeping the genome pristine. The obvious benefit to preventing somatic mutations is the maintenance of the genetic code, upon which all cells rely to function normally. The costs of such perfect genome maintenance would comprise the energy needed to drive such vast surveillance, and the extension of the cell cycle resulting in slowed growth, healing, and a reduced ability to maintain homeostasis. Ren et al. review the challenges to studying somatic mutations, and discuss advances made using somewhat underutilized methods of somatic mutation analysis, such as clone sequencing and duplex sequencing. Finally, the authors discuss the potential consequences of the accumulation of somatic mutations during aging, including the destabilization of gene regulatory networks due to mutations in regulatory and coding regions. They also point out that somatic mutations are causal in not only cancer but also of non-cancerous clonal expansions that can be detrimental to tissue function.

Sanchez-Contreras and Kennedy review the complicated relationship between mitochondrial somatic mutations and aging. Mitochondria are the primary site of energy generation in the cell, and as such, are a focal point for the generation of reactive oxygen species (ROS), which can damage DNA and cause somatic mutations. While somatic SNVs in the mitochondrial genome increase with human age, somatic mtDNA deletions seem more common. One such deletion, the 4,977bp so-called “common deletion”, is so prevalent across tissues that it has been proposed as a biomarker to distinguish physiological from accelerated aging. mtDNA mutation rates are higher in neurodegenerative disease, and, interestingly, DNA polymerase-gamma (POLG), a mitochondrial DNA repair factor, is a risk gene for Parkinson’s disease. POLG loss in mouse, fly, and worm models results in progeria, suggesting somatic mtDNA mutations can directly cause aging; however, mtDNA replication is slowed in the absence of POLG, complicating the ability to unambiguously link mtDNA damage to aging. The source of age-related mtDNA mutations is up for debate, with DNA damage or replication errors being the two most prominent possible causes. Interestingly, 8-oxo-7,8-dihydroguanine (8-oxoG), the most prominent and well-studied DNA lesion caused by oxidative DNA damage, seems not to be directly related to mtDNA somatic mutations, suggesting that other oxidative metabolic byproducts of oxidative phosphorylation, or ROS-independent mechanisms, drive mtDNA mutation rates.

Walsh et al. present a summary of a March 2021 workshop on clonal hematopoiesis convened by the National Institute on Aging involving experts from academia, industry, and government. A substantial number (15%–20%) of healthy individuals age 70 + display at least one cancer-associated somatic mutation at ≥ 2% variant allele frequency in nucleated blood cells. This phenomenon, known as clonal hematopoiesis of indeterminate potential (CHIP), occurs most commonly due to pro-growth mutations in hematopoietic stem/progenitor transcriptional regulators such as DNMT3A, TET2, or ASXL1. CHIP is associated with a variety of adverse outcomes. Individuals with CHIP show an increased “epigenetic age”, a metric derived from analysis of DNA methylation changes during aging. Further, CHIP+ individuals are at increased risk of specific diseases, including atherosclerotic cardiovascular disease, heart failure, and venous thrombosis. CHIP is also associated with increased inflammation. Taken together, it is thus not surprising that CHIP is modestly associated with increased all-cause mortality.

Ziegenfuss and Lodato review the dual role of 8-oxoG in the brain during life. As discussed in other reviews in this Research Topic, oxidative DNA damage is a source of at least some age-related somatic mutations. 8-oxoG is the product of oxidative attack on guanine nucleotides. 8-oxoG is mutagenic because it can pair with adenine during DNA replication to generate G/C>T/A somatic mutations. Numerous repair pathways exist to mitigate this mutagenic phenomenon. The base excision repair proteins OGG1 and MUTYH are responsible for removing 8-oxoG and 8-oxoG:A mispairs from the DNA, respectively. Nucleotide excision repair can also clear 8-oxoG lesions from the genome where OGG1 and MUTYH fail. This raises the question: how does 8-oxoG result in mutation in the context of multiple redundant repair mechanisms that exist to prevent it from doing so? The answer may be in an idea called antagonistic pleiotropy, the phenomenon by which the same factor can have opposite effects depending on life stage. Indeed, 8-oxoG plays a role in the epigenetic regulation of the genome, with local 8-oxoG rates in DNA influencing DNA methylation and, therefore, gene expression levels of nearby genes. Thus, some low level of somatic mutation accumulation due to 8-oxoG may be the cost of using this lesion to regulate gene expression levels.

## Future perspectives

Each of the above reviews raises interesting and worthwhile future directions for their respective areas of somatic mutation research. Based on the last several years of research, the steady accumulation of somatic mutations in virtually all cells of the body is at this point an unquestioned fact. In our opinion, going forward the first and foremost question in this field is: What role does increasing genome mosaicism play in aging and age-related disease? Specifically, outside of inducing cancer and other non-cancerous clonal expansions, does an increased burden of somatic mutations in the body impact aging or age-related disease? Another major set of questions relates to the causes of somatic mosaicism during aging. What are the cell-type and tissue-specific drivers of somatic mutation? Are they druggable, meaning this process can be controlled? Would reducing the somatic mutation rate reduce the decline in function and increase in disease incidence associated with aging? We hope these and other questions in the field are pursued in the near future.

## References

[B1] Biswas K. Alexander K. Francis M. M. (2022). Reactive oxygen species: Angels and demons in the life of a neuron. NeuroSci 3, 130–145. 10.3390/neurosci3010011 PMC11523706 39484669

[B2] Dou Y. Gold H. D. Luquette L. J. Park P. J. (2018). Detecting somatic mutations in normal cells. Trends Genet. 34, 545–557. 10.1016/j.tig.2018.04.003 29731376 PMC6029698

[B3] Franco I. Fernandez-Gonzalo R. Vrtacnik P. Lundberg T. R. Eriksson M. Gustafsson T. (2019). Healthy skeletal muscle aging: The role of satellite cells, somatic mutations and exercise. Int. Rev. Cell Mol. Biol. 346, 157–200. 10.1016/bs.ircmb.2019.03.003 31122394

[B4] Jaiswal S. Ebert B. L. (2019). Clonal hematopoiesis in human aging and disease. Science 366, eaan4673. 10.1126/science.aan4673 31672865 PMC8050831

[B5] Koh G. Degasperi A. Zou X. Momen S. Nik-Zainal S. (2021). Mutational signatures: Emerging concepts, caveats and clinical applications. Nat. Rev. Cancer 21, 619–637. 10.1038/s41568-021-00377-7 34316057

[B6] Miller M. B. Reed H. C. Walsh C. A. (2021). Brain somatic mutation in aging and alzheimer's disease. Annu. Rev. Genomics Hum. Genet. 22, 239–256. 10.1146/annurev-genom-121520-081242 33979534 PMC8612367

[B7] Smith A. L. M. Whitehall J. C. Greaves L. C. (2022). Mitochondrial DNA mutations in ageing and cancer. Mol. Oncol. 16, 3276–3294. 10.1002/1878-0261.13291 35842901 PMC9490137

[B8] Valecha M. Posada D. (2022). Somatic variant calling from single-cell DNA sequencing data. Comput. Struct. Biotechnol. J. 20, 2978–2985. 10.1016/j.csbj.2022.06.013 35782734 PMC9218383

[B9] Wang Y. Sano S. Ogawa H. Horitani K. Evans M. A. Yura Y. (2022). Murine models of clonal haematopoiesis to assess mechanisms of cardiovascular disease. Cardiovasc Res. 118, 1413–1432. 10.1093/cvr/cvab215 34164655 PMC9075001

